# Immigrant and Border Infectious Disease Concerns for Women^1^

**DOI:** 10.3201/eid1011.040624_13

**Published:** 2004-11

**Authors:** Alexandra Levitt, Michelle Weinberg, Margarita Irizarry De La Cruz, Gloria A. Kilanko

**Affiliations:** * Centers for Disease Control and Prevention, Atlanta, Georgia, USA;; † Women Watch Afrika, Inc., Decatur, Georgia, USA

**Keywords:** disease surveillance, healthcare, hepatitis, infectious disease, immigrants, rubella listeriosis

Immigrant women may be at risk for diseases such as rubella (due to lack of vaccination) or listeriosis (due to food preparation practices). They may also face a variety of cultural and social barriers that prevent them from obtaining adequate health care.

## Mexico-U.S. Border Infectious Disease Surveillance

The Border Infectious Disease Surveillance (BIDS) project demonstrates that a binational effort with local, state, and federal participation can overcome political, cultural, and infrastructural barriers to create a regional system for monitoring infectious diseases that crosses an international border. Established in 1997 by the Mexican Secretariat of Health, the U.S. Centers for Disease Control and Prevention, and border health officials, BIDS has created a sentinel surveillance network that monitors hepatitis and febrile exanthems at 13 clinics on both sides of the Mexico-U.S. border. As part of this effort, BIDS has developed binational surveillance protocols, trained surveillance coordinators, established serologic testing at Mexican border laboratories, and developed agreements for reporting and data-sharing. In 1999, BIDS facilitated a binational vaccination campaign against rubella, a disease that causes birth defects when it infects pregnant women.

## Knowledge, Attitudes, and Practices among Immigrant Hispanic Women Regarding Consumption of High-Risk Foods

Hispanic/Latino immigrants are at high risk for listeriosis, a disease that can cause severe pregnancy complications, including miscarriage, stillbirth, uterine infection, premature labor, and death in the newborn period. The goal of CDC’s Futura Mama program is to assess community attitudes and knowledge about unpasteurized milk products (a common vehicle for listeriosis) and use this information to develop culturally sensitive disease prevention strategies. Eight focus groups were conducted in a Hispanic/Latino immigrant community in Georgia. Most participants reported regular consumption of homemade cheese and thought that unpasteurized milk and milk products are healthier and tastier than store-bought products. None of the participants were aware of listeriosis, of other infections related to unpasteurized milk products, or of the association between infection and pregnancy complications. Women <32 years of age were more likely than those >32 years to accept the idea that health risks are associated with unpasteurized milk products. The study concluded that public health messages delivered through in-person formats (workshops and discussion groups) and mass media may be effective in reducing the risk of listeriosis in Hispanic/Latino communities.

## Immigrant Women and Challenges of Infectious Disease

Immigrant women must overcome cultural, linguistic, and economic barriers to obtain basic healthcare services for themselves and their families. Cultural barriers may include traditional practices that give boys and men preference in access to medical services or that stigmatize a woman who is known to be sick. A woman may be dependent on her husband to pay her medical bills, or she may not be allowed to see a male physician on the basis of her religion. In addition, she may be afraid to disclose the source of a sexually transmitted infection, especially if it is the result of sexual violence. Healthcare programs can help immigrant women by working with community-based organizations to develop procedures and attitudes that are more gender-sensitive and immigrant-friendly. Primary care programs can hire and train culturally-diverse and multilingual staff members who are sensitive to different cultures, traditions, and religions. They can develop partnerships with other immigrant-serving agencies and find ways to make healthcare services more attractive and accessible to immigrant women.

Nongovernmental organizations that address the health concerns of Hispanic and Latino immigrants might join forces with those that address the concerns of immigrants from Africa and Asia to form a nationwide immigrant health coalition, which represents all U.S. immigrants in policy-making situations.

